# BACH2 in TRegs Limits the Number of Adipose Tissue Regulatory T Cells and Restrains Type 2 Immunity to Fungal Allergens

**DOI:** 10.1155/2022/6789055

**Published:** 2022-08-05

**Authors:** Amanda Contreras, Darin L. Wiesner, Brock Kingstad-Bakke, Woojong Lee, John P. Svaren, Bruce S. Klein, M. Suresh

**Affiliations:** ^1^Department of Pathobiological Sciences, University of Wisconsin-Madison, Madison, 53706 WI, USA; ^2^Department of Medical Microbiology and Immunology, University of Wisconsin-Madison, Madison, 53706 WI, USA; ^3^Department of Comparative Biosciences, University of Wisconsin-Madison, Madison, 53706 WI, USA

## Abstract

FoxP3+ regulatory T cells (Tregs) are essential for self-tolerance and moderating tissue-damaging inflammation. Tregs that develop and mature in the thymus are classified as central Tregs or effector Tregs based on whether Tregs predominately inhabit secondary lymphoid organs (central Tregs) or tissues (effector Tregs). By generating mice that are conditionally deficient for Bach2 in peripheral Tregs, we have examined the role of Bach2 in regulating Treg homeostasis and effector functions. Unlike global and T cell-specific Bach2-deficient mice, Treg-specific Bach2 ablation did not result in unprovoked T_H_2 inflammation in the lungs. However, Bach2 deficiency in Tregs led to augmented expressions of IRF4, BATF, and GATA3 and a significant increase in the accumulation of ST2 (IL-33R)^+ve^ effector Tregs in the spleen and visceral adipose tissue (VAT) but not in the lungs. Enhanced Bach2-deficient Treg numbers in VAT was not linked to hyperresponsiveness to exogenous IL-33 in vivo. Most strikingly, Treg-specific Bach2 deficiency resulted in enhanced fungal protease-induced Type 2 allergic inflammation in the lungs, with no detectable effects on Type 1 responses to systemic or respiratory viral infections. In summary, we ascribe vital roles for Bach2 in peripheral Tregs: as a transcriptional checkpoint to limit precocious differentiation into effector Tregs in lymphoid tissues and as a regulator of the functional program that restrains Type 2 but not Type 1 inflammation in lungs. Results presented in this manuscript implicate dysregulated Tregs in the pathogenesis of airway hypersensitivities, asthma, and other allergic disorders.

## 1. Introduction

Foxp3+ regulatory T (Treg) cells are a vital T-cell subset that enforce self-tolerance and mitigate inflammatory pathology during immune responses to foreign antigens [1]. Peripheral Treg cells are divided into two distinct subtypes based on their anatomical distribution and function: central (cTregs) and effector Tregs (eTregs) [2, 3]. The majority of Treg cells in the secondary lymphoid tissues are cTregs that express high levels of lymphoid homing receptors CD62L and CCR7 and have trafficking patterns similar to naïve conventional T cells. By contrast, eTregs constitute a small fraction of Tregs in the lymphoid tissues, but the majority of Tregs in the peripheral tissues. Unlike cTregs, the eTregs display an activated effector phenotype (e.g., low expression levels of CD62L and CCR7, but high levels of CD44, inducible T cell costimulatory [ICOS], glucocorticoid-induced tumor necrosis factor receptor [GITR], and KLRG-1) with trafficking patterns similar to conventional effector T cells (i.e., traffic between blood and peripheral non-lymphoid tissues) [4, 5]. These Foxp3+ eTregs have been further classified into T_H_1-Tregs, T_H_2-Tregs, and T_H_17-Tregs depending upon their polarity and co-opted expression of T-bet, GATA-3, and STAT-3 transcription factors, respectively [6]. This pattern of polarized differentiation endows each eTreg subset with distinct migratory and functional properties tailored to specifically balance inflammatory responses driven by T_H_1, T_H_2, or T_H_17 effector T cells. While IL-2R signaling is clearly necessary for eTreg cell differentiation [7–9], activation and polarization of cTregs into eTreg cell subsets are driven by TCR signaling and the inflammatory milieu in the peripheral tissues [10–13].

Tissue Tregs are a specialized subset of effector Tregs that reside in non-lymphoid tissues [14] and display characteristics including expression of transcription factors and homing or effector molecules that allow them to specialize for their specific tissue environment [14]. The IL-33 receptor ST2 thus far appears to be a universal feature of tissue Tregs that distinguishes them from classical lymphoid-organ Tregs [14]. While it is known that BATF and IRF4 are required for the induction of key components of the effector Treg transcriptional program, it has recently been demonstrated that BATF and IRF4 are required for the maintenance of tissue Tregs and expression of ST2 [15]. Furthermore, IL-33 induces GATA-3 phosphorylation, which subsequently binds to the ST2 locus to promote ST2 gene expression [16].

The transcriptional mechanisms that actively maintain the “quiescence” of cTregs or oppose the premature differentiation of eTregs or tissue Tregs from cTregs are not well understood. Bach2 is a transcription repressor that plays an integral role in the maintenance of T cell quiescence, differentiation, and generation of memory T cells [17–19]. Using global Bach2-deficient mice, we and others have shown that Bach2 plays crucial roles in the differentiation and competitive fitness of Tregs and protects against fatal T_H_2-driven eosinophilic crystalline pneumonia [20, 21]. Further, it has been reported that ablation of Bach2 in all T cells leads to dysregulated T_H_2 immunity and pulmonary inflammation in mice [22–24]. Until recently, Treg-specific role of Bach2 in maintaining the homeostasis of peripheral Tregs or suppressing T_H_1/T_H_2 immunity was unknown. Two reports have since shown that Bach2 restrains the differentiation of eTregs by repressing genes regulated by transcription factors such as IRF-4 [25–27]. Here, we report that ablation of Bach2 in peripheral Tregs leads to enhanced differentiation of effector/tissue Tregs in the lymphoid tissues, increased numbers of visceral adipose tissue (VAT) Tregs, and increased activation of T cells in response to normal homeostatic cues. Further, we show that loss of Bach2 in Tregs does not affect the development of T_C_1/T_H_1 cells during mucosal or systemic viral infections but instead augments the differentiation of IL-5 and IL-13-producing T_H_2 cells during fungal allergen-induced inflammation in the lungs. These findings provide new insights into the Treg-specific roles for Bach2 in (i) regulating the homeostasis of splenic and VAT eTregs and (ii) restraining fungal protease-induced Type 2 immunity but not virus-induced Type 1 immunity.

## 2. Materials and Methods

### 2.1. Mice

Mice with a FoxP3+ Treg-specific deletion of Bach2 were generated by breeding floxed Bach2 mice [19] with B6.129(Cg)-*Foxp3^tm4(YFP/icre)Ayr^*/J mice [28] to create Bach2^loxp/loxp^FoxP3-Cre mice. Either littermate WT or C57BL/6 J mice were used as controls. The mice used in these studies were housed in specific-pathogen-free conditions in University of Wisconsin-Madison animal facilities. All animal experiments were conducted in accordance with approved protocols of the institutional animal care committee.

### 2.2. Viral Infections and Protease Treatment

The Armstrong strain of lymphocytic choriomeningitis virus (LCMV) was administered intraperitoneally at 2×10^5^ plaque-forming units (PFU) per mouse to six- to eight-week-old mice; virus-specific T cell responses in spleen were quantified at day 8 after LCMV infection. The influenza virus strain A/PR/8/34 H1N1 (PR8) was administered intranasally at 100 PFU per mouse. Mice were euthanized, and lungs were harvested 10 days after PR8 infection. For fungal protease treatment, 10-week-old mice were anesthetized to receive intratracheal doses of 25 ug of fungal protease from *Aspergillus melleus* (Sigma #P4032) on days 1, 2, 7, and 14. On day 15 after the initial treatment, mice were injected with 3 ug of anti-CD45 antibody intravenously immediately prior to euthanasia and lung harvest.

### 2.3. IL-33 In Vivo Administration

Mice received intraperitoneal injections of 0.5 ug of recombinant murine IL-33 (Peprotech) or PBS on days 0, 2, 4, and 6 [29]. On the 8th day of IL-33 treatment, mice were euthanized to harvest lungs, VAT, and spleens.

### 2.4. IL-33 In Vitro Treatment

Spleens from naïve Bach2^loxp/loxp^FoxP3-Cre and WT mice were processed into single cell suspensions and were plated at a concentration of 5 × 10^5^ cells/well in a 96-well plate. Prior to plating cells, wells were coated with 1ug/mL anti-CD3. Cells were cultured for 72 hours with IL-2 (10 ng/mL) (BD Biosciences) and anti-CD28 (2ug/mL) in either the presence or absence of 1 ng/mL IL-33. Cells were then collected and processed for flow cytometry.

### 2.5. Flow Cytometry

Single-cell suspensions of mononuclear cells from lung, VAT, and spleen were prepared using standard procedures. Lung and VAT were digested in 2 mg/mL Collagenase D (Sigma) for 30 minutes while rotating at 37 °C. The digested tissues were then homogenized using the GentleMACS Dissociator (Miltenyi). Single-cell suspensions were first stained for viability with a LiveDead stain (eBioscience). Cells were then resuspended and stained with antibodies diluted in a staining buffer of either 2% BSA in PBS or in Brilliant Stain Buffer (BD Biosciences), depending on the combination of antibodies used. The fluorochrome-labeled antibodies that were used against cell-surface antigens include the following: CD8, CD4, CD25, CD44, CD62L, CD127, CD69, GITR, CTLA-4, KLRG-1, CCR7, CD103, CXCR3, ST2, CD27, CD11b, CD90.2, CD64, Siglec-F, PD-L1, CD19, CD11c, Ly6G, *γδ* TCR, and TCR-*β*. Fluorochrome-labeled antibodies that were used against intracellular antigens include the following: IFN-*γ*, IL-4, IL-5, IL-13, IL-17A, BATF, IRF4, GATA3, Foxp3, Ki-67, Eomes, Tbet, and Helios. These antibodies were purchased from BD Biosciences (San Jose, CA), Biolegend (San Diego, CA), or eBioscience (San Diego, CA). Fluorochrome-conjugated tetramers for LCMV epitopes (D^b^/NP396, D^b^/GP33, I-A^b^/GP66) and for PR8/H1N1 epitopes (D^b^/NP366, D^b^/PA224, I-A^b^/NP311) were provided by the NIH Tetramer Core Facility (Emory University, Atlanta, GA). Samples were acquired with a BD LSRFortessa (BD Biosciences), and resulting data was analyzed with FlowJo software (TreeStar, Ashland, OR).

### 2.6. Intracellular Cytokine Staining

To induce cytokine production in cells for intracellular cytokine staining, cells were stimulated directly ex vivo with human recombinant IL-2 (10 U/well) (BD Biosciences) and the epitope peptide at 0.1ug/mL. Depending on the experiment, cells were either stimulated with PMA/Ionomycin (Tonbo Biosciences) or LCMV peptides (NP396, GP33, and GP276) or PR8/H1N1 peptides (NP366, PA224, and NP311) (thinkpeptides, ProImmune Ltd.). Cells undergo 5 hours of stimulation at 37 °C in the presence of brefeldin A (1ug/mL, GolgiPlug, BD Biosciences). After stimulation, cells were stained for cell surface antigens, fixed, and permeabilized using the Cytofix/Cytoperm kit (BD Sciences). After fixation/permeabilization, cells were incubated with fluorochrome-labeled antibodies targeted against cytokines. For the fungal protease studies, cells extracted from the lungs were restimulated directly ex vivo with 100 ug/well of heat inactivated fungal protease for 6 hours. Brefeldin A was added for the last 4 hours of the restimulation. The cells were then fixed, permeabilized, and stained for intracellular cytokine expression as described above.

### 2.7. Intracellular Staining for Transcription Factors and Ki-67

To stain for intracellular proteins such as transcription factors and Ki-67, cells were fixed and permeabilized with the FoxP3 Staining Kit (eBioscience) using the manufacture's protocol. After fixation/permeabilization, cells were incubated with fluorochrome-labeled antibodies targeted against transcription factors and Ki-67. After staining, cells were analyzed with a BD LSRFortessa flow cytometer.

### 2.8. Statistical Analyses

Data statistics were calculated with Prism software (GraphPad Software, La Jolla, California, USA). Student's two-tailed *t*-test and one-way ANOVA analyses were used to calculate the statistical significance of differences between groups, and significance was defined at *p* < 0.05.

## 3. Results

### 3.1. Bach2 Restrains the Activation and Differentiation of Effector Tregs and Maintains Homeostasis of Naïve and Activated/Memory T Cells

**Figure 1:**
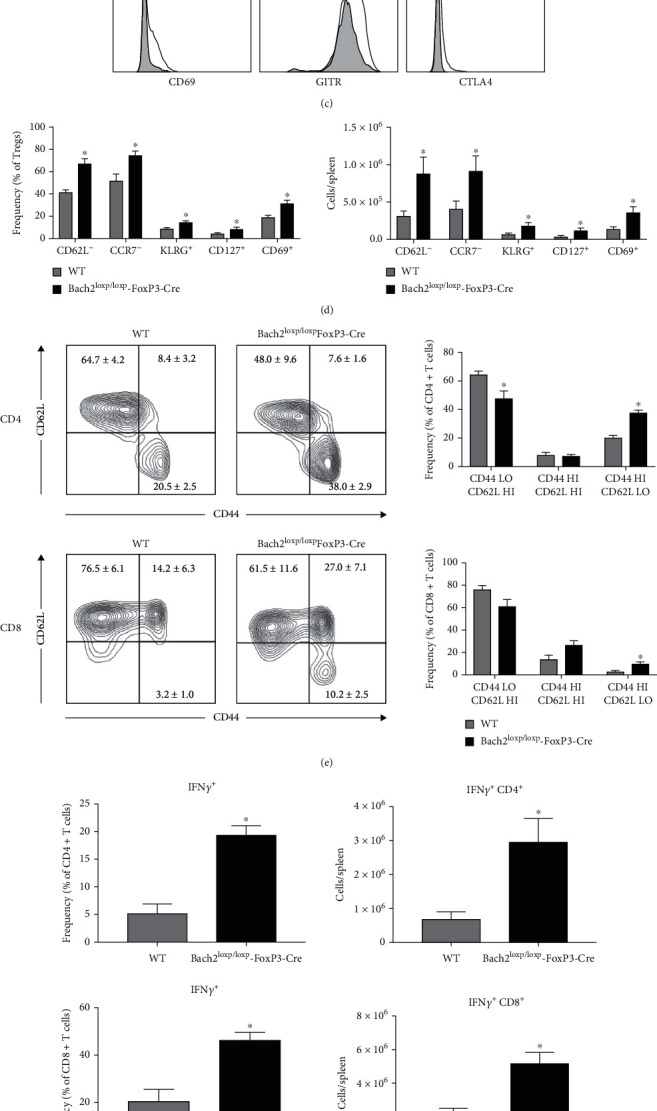
Bach2 regulates classical and regulatory T cell homeostasis. Splenocytes from naïve WT and Bach2^loxp/loxp^FoxP3-Cre mice were stained for intracellular and extracellular antigens and analyzed by flow cytometry. (a) Frequency and (b) number of Foxp3^+ve^ Treg cells. (c) Histograms are gated on Foxp3^+ve^ Treg cells and show staining for the indicated markers in WT (shaded) and Bach2^loxp/loxp^FoxP3-Cre mice (line) mice. (d) Frequency and numbers of Tregs expressing the indicated molecules. (e) Activation of classical T cells was analyzed by staining with anti-CD44 and anti-CD62L; contour plots are gated on CD4 or CD8 T cells. (f), (g) Splenocytes were stimulated for 5 hrs in vitro with PMA and ionomycin in the presence of Brefeldin A, and cytokine production by CD4 and CD8 T cells was measured by intracellular staining for (f) IFN-*γ* and (g) IL-13 and IL-17A. This experiment was repeated three times with similar results, 4-5 mice per group. ∗*p* < 0.05.

We and others have previously reported that global Bach2 deficiency leads to aberrant differentiation of Tregs and activated/memory phenotype of peripheral T cells [20, 21]. To investigate the effect of conditional Bach2 deficiency in mature Tregs, we bred floxed Bach2 mice with Rudensky's Foxp3-Cre mice to generate the Bach2^loxp/loxp^FoxP3-Cre mice. Loss of Bach2 in Tregs did not alter the frequency or total numbers of foxp3^+ve^ Tregs in spleen, which suggested that Bach2 is not required for the development and/or maintenance of these cells (Figures 1(a) and 1(b)). Next, we assessed whether Treg-specific Bach2 deficiency dysregulated the homeostasis of naïve and activated/effector Tregs. Notably, Bach2-deficient Tregs exhibited enhanced expression of CD44, CD127, CD69, and GITR, which was strongly suggestive of skewed differentiation favoring an effector Treg phenotype (Figure 1(c)) [4, 5]. Clearly, there was a significant increase in the percentages and numbers of the activated/effector (CD44^HI^/CD62L^LO^/CCR7^LO^) Bach2-deficient Tregs, as compared to their WT counterparts (Figure 1(d)). Further, there were increased frequencies and total numbers of Bach2-deficient Tregs that expressed high levels of KLRG1, CD127, and CD69.

Next, we investigated whether loss of Bach2 in Tregs affected the homeostasis of classical naïve and activated/memory T cells in *trans*. We found an increase in the percentages of CD4 and CD8 T cells with an activated/effector phenotype (CD44^HI^/CD62L^LO^) in the spleens of Bach2^loxp/loxp^FoxP3-Cre mice compared to WT counterparts (Figure 1(e)). In addition, we observed a decrease in the percentages of CD4 T cells with a naïve phenotype (CD44^LO^/CD62L^HI^) (Figure 1(e)). Next, we were interested in whether Bach2-deficient Tregs altered the production of cytokines from conventional T cells. Splenic CD8 and CD4 T cells had a substantial increase in the percentages and total numbers of cells that produced IFN*γ* in the Bach2^loxp/loxp^FoxP3-Cre mice compared to WT mice. Additionally, CD4 T cells from Bach2^loxp/loxp^FoxP3-Cre mice displayed higher frequencies of IL-13 and IL-17A-producing cells as well as total cells that produced IL-17A (Figures 1(f) and 1(g)). These augmented levels of cytokine-producing cells suggest that Bach2-deficient Tregs are unable to repress the activation and differentiation of T_H_1 (IFN*γ*), T_H_2 (IL-13), and T_H_17 (IL-17A) effector CD4 T cells. Taken together, these data suggested that Bach2 plays an essential role in maintaining quiescence/naivety by restraining the differentiation of naïve Tregs into effector Tregs. Furthermore, loss of Bach2 expression in Tregs led to the development of activated/memory T cells, which suggests that the ability of Tregs to limit activation of classical T cells requires Bach2 expression in Tregs.

### 3.2. Bach2 Represses Tissue Treg-like Cell Differentiation in Secondary Lymphoid Tissues

**Figure 2:**
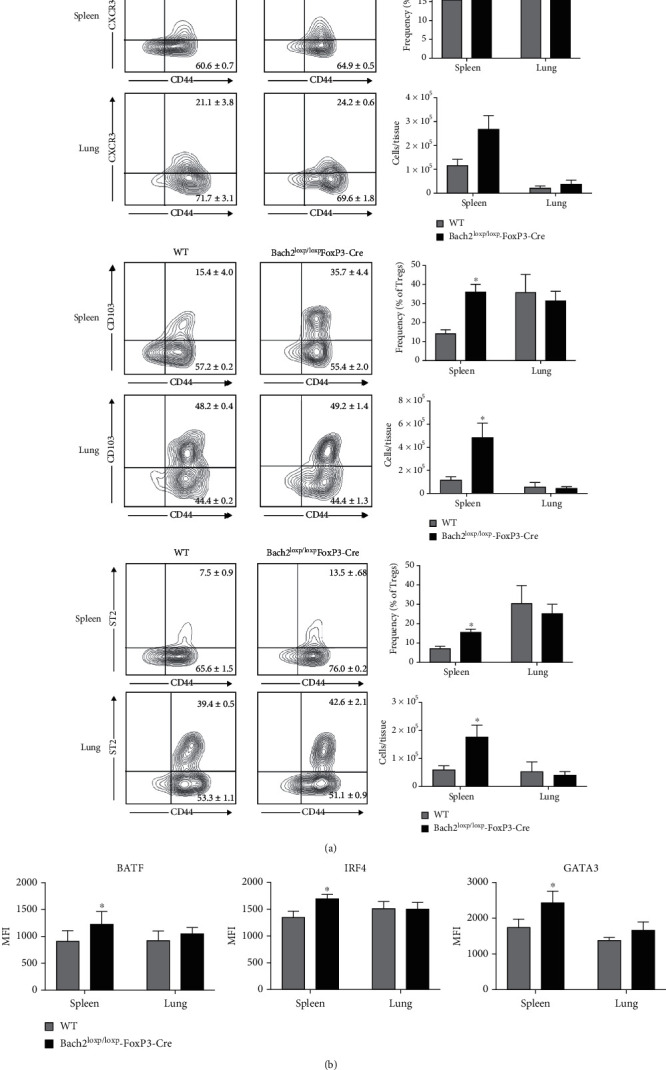
Bach2 restrains tissue regulatory T cell differentiation in the spleen. Spleens and lungs were collected from naïve WT and Bach2^loxp/loxp^FoxP3-Cre mice and Tregs were analyzed by flow cytometry. (a) Frequency and number of Foxp3^+ve^ Tregs in lung and splenic Treg cells that expressed CXCR3, CD103, or ST2. (b) Data shows MFI levels for BATF, IRF4, and GATA3 staining in Foxp3^+ve^ Tregs. This experiment was repeated three times with similar results, 4-5 mice per group. ∗*p* < 0.05.

Apart from the well-defined subsets of naïve and effector Tregs in secondary lymphoid tissues, recent work has identified a distinct subset of Tregs termed as tissue Tregs that can be found in non-lymphoid tissues such as lungs, skeletal muscle, and lamina propria [14]. While these tissue Tregs display the prototypical effector surface phenotype markers such as CXCR3 and CD103 and express transcription factors BATF, IRF4, and GATA3, they possess certain properties that make each tissue Treg population unique. However, tissue Tregs possess a distinguishing surface marker that has been found on all tissue-residing Tregs populations currently examined: ST2. ST2 is a receptor for IL-33 and is expressed on Tregs that preferentially accumulate in non-lymphoid tissues [30, 31]. It was of interest to determine whether Bach2 deficiency dysregulated the differentiation of tissue Tregs in lymphoid tissues. As expected, a modest fraction of Tregs expressed CD103, CXCR3, and ST2 in spleens of WT mice. Surprisingly, however, we found that Bach2 deficiency in Tregs led to a substantive increase in the frequencies of CD103^+ve^, CXCR3^+ve^, and ST2^+ve^ Tregs in spleen, as compared to their WT counterparts. The total numbers of CD103^+ve^ and ST2^+ve^ Tregs were significantly higher in the spleens of Treg-specific Bach2-deficient mice than in WT mice (Figure 2(a)). It is possible that the increased numbers of tissue Treg-like cells in the spleen of Treg-specific Bach2-deficient mice could result from defects in trafficking of these cells to non-lymphoid tissues. To address this possibility, we quantified Tregs in lungs and liver of Treg-specific Bach2-deficient mice. Data in Figure 2 show that the percentages and numbers of Tregs in lungs and liver (data not shown) of Treg-specific Bach2-deficient mice were comparable to those in WT mice. Therefore, it is likely that tissue Tregs are accumulating in spleen of Treg-specific Bach2-deficient mice due to increased differentiation and not driven by defective trafficking of these cells from spleen to non-lymphoid tissues.

We next investigated whether Bach2 deficiency-induced enhancement in the development of ST2^+ve^ tissue Tregs was associated with altered expression of tissue Treg fate determining transcription factors BATF, IRF4, and GATA3. Indeed, we found statistically significant increases of BATF, IRF4, and GATA3 expression in Bach2-deficient Tregs compared to their WT counterparts only in spleen, but not in lungs (Figure 2(b)). Overall, our studies have revealed an unexpected increase in the ST2^+^ CD103^+^ CXCR3^+^ tissue Treg-like cells in the spleen of mice harboring a Treg-specific Bach2 deletion. These data suggest that Bach2 exerts a transcriptional block in the differentiation of tissue Treg-like cells in the lymphoid tissues.

### 3.3. Bach2 Limits the Numbers of Visceral Adipose Tissue Tregs

**Figure 3:**
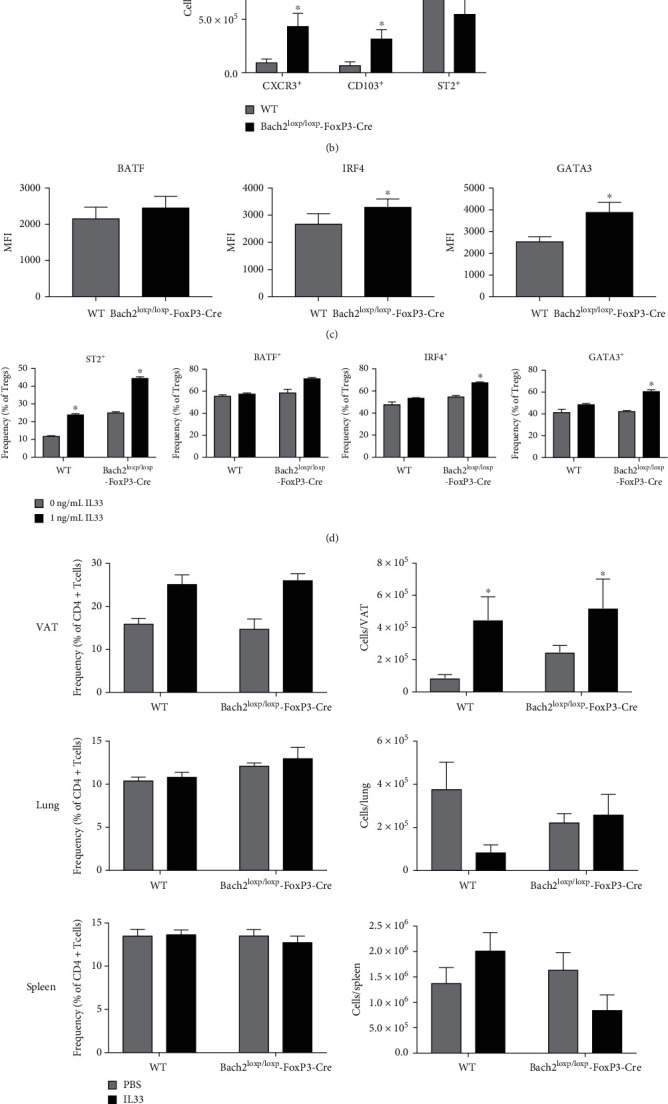
Bach2 limits the size of tissue regulatory T cell population in the VAT. Mononuclear cells isolated from the VAT of WT and Bach2^loxp/loxp^FoxP3-Cre mice were analyzed by flow cytometry. Tregs in the VAT of naïve WT and Bach2^loxp/loxp^FoxP3-Cre mice were analyzed for (a) their frequency and numbers, (b) tissue Treg marker expression, and (c) MFI levels for BATF, IRF4, and GATA3. (d) Splenocytes from naïve WT and Bach2^loxp/loxp^FoxP3-Cre mice were stimulated in vitro with anti-CD3, anti-CD28, and IL-2 in the presence of absence of IL-33 for 72 hrs. Bar graphs display frequencies of ST2^+ve^ and BATF^+ve^, IRF4^+ve^, or GATA3^+ve^ Tregs. (E-H) WT and Bach2^loxp/loxp^FoxP3-Cre mice received four injections of IL-33, and the indicated tissues were analyzed on day 8. (e) Frequency and number of Treg cells in the VAT, lungs, and spleen. (f) Number of CXCR3-, CD103-, and ST2-positive Tregs in the VAT. This experiment was repeated two times with similar results, 5 mice per group. ∗*p* < 0.05.

Tissue Tregs have been most thoroughly characterized in the VAT, and transcription factors BATF and IRF-4 bind to the ST2 promoter and promote ST2 expression [15]. Since Bach2-deficient Tregs express elevated levels of ST2, BATF, and IRF-4 (Figure 2), it was of interest to investigate whether Bach2 regulated the homeostasis of VAT Tregs. Analysis of Tregs in VAT showed that Bach2 deficiency led to a significant increase in the numbers of CD103^+ve^ and CXCR3^+ve^ Tregs in VAT (Figure 3(b)); the numbers of ST2^+ve^ Tregs were not significantly altered in VAT of Treg-specific Bach2 deficient mice. Next, we examined whether Bach2 deficiency-induced increased numbers of VAT Tregs was associated with elevated expression of transcription factors that drive the VAT Treg program. We found that Bach2-deficient VAT Tregs exhibited significantly higher levels of GATA3 and IRF4 compared to WT Tregs (Figure 3(c)).

ST2 is the cellular receptor for IL-33, and it is known that ST2 and IL-33 are required for the development of VAT Tregs [15]. Mechanistically, IL-33 enhances ST2 expression by inducing GATA-3 phosphorylation and subsequent recruitment of GATA-3 to the ST2 locus. Because Bach2-deficient Tregs have higher expression of ST2 in the spleen and express elevated levels of GATA3 and IRF4, we explored whether Bach2-deficient Tregs are more poised to the effects of IL-33 and therefore more readily differentiate into tissue Tregs. As expected, in vitro exposure of WT splenic Tregs to IL-33 induced ST2 expression in a fraction of Tregs (Figure 3(d)). While the percentages of ST2-expressing Tregs were already higher among untreated Bach2-deficient Tregs, IL-33 treatment further increased the percentages of ST2^+ve^ Tregs (Figure 3(d)). The magnitude of ST2 induction was comparable in WT and Bach2-deficient Tregs, but the IL-33-induced expressions of GATA3, IRF4, and BATF were greater in Bach2-deficient Tregs, as compared to those in WT Tregs (Figure 3(d)), which suggest that Bach2 restrains IL-33-induced expression of these transcription factors in Tregs.

Exogenous IL-33 administration has been reported to drive the expansion of VAT Tregs in vivo [15]. Here, we investigated whether Tregs from WT or Bach2-deficient Tregs mice respond differently to IL-33 treatment in vivo. IL-33 treatment significantly augmented the numbers of Tregs only in the VAT, but not in the spleen or lungs (Figure 3(e)) of both WT and Bach2^loxp/loxp^FoxP3-Cre mice. The IL-33-responsive population of Tregs included CD103^+ve^, CXCR3^+ve^, and ST2^+ve^ Tregs in both groups of mice (Figure 3(f)). Mechanistically, IL-33-driven expansion of Tregs in VAT was associated with elevated percentages of proliferating Ki67^+ve^ Tregs (not shown) in both WT and Bach2^loxp/loxp^FoxP3-Cre mice. As compared to PBS-treated controls, the magnitude of increase in the number of Tregs was similar for WT and Bach2^loxp/loxp^FoxP3-Cre mice. Taken together, data in Figure 3 strongly suggest that Bach2 deficiency did not alter the IL-33-driven proliferative expansion of VAT Tregs.

### 3.4. Bach2-Deficiency in Tregs Does Not Alter the Development of T_H_1 or T_C_1 Cells during an Acute Viral Infection

**Figure 4:**
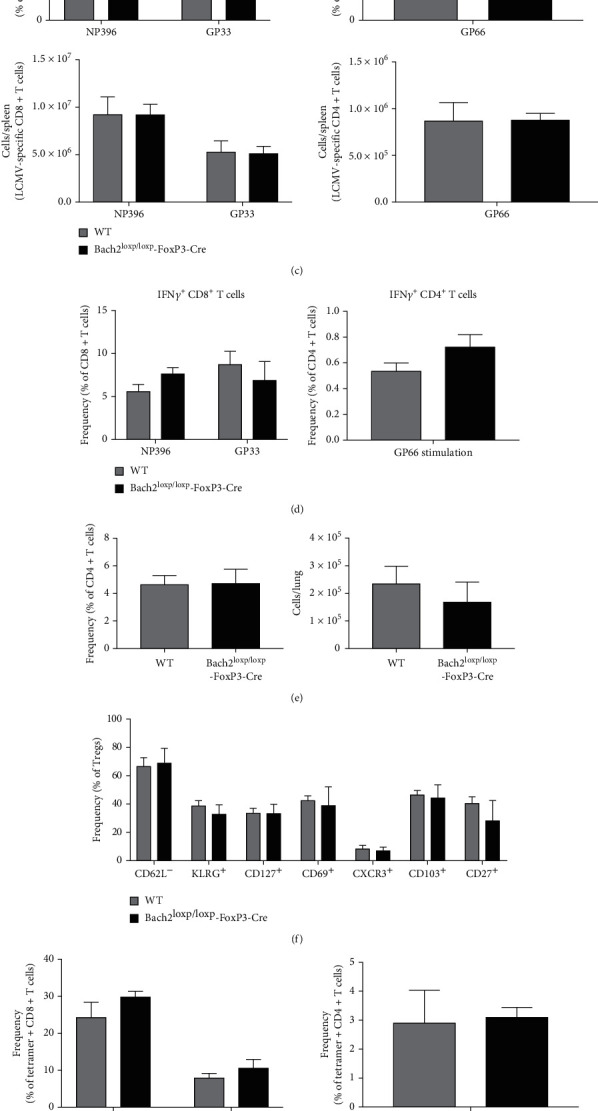
Bach2-deficiency in Tregs does not affect systemic or mucosal Type 1 immunity. WT and Bach2^loxp/loxp^FoxP3-Cre mice were infected with (a-d) LCMV-Armstrong or (e-h) PR8/H1N1 influenza virus, and Tregs were analyzed by flow cytometry. (a) Frequency and numbers of splenic Tregs in LCMV-infected mice. (b) Frequency of effector Treg subsets in the spleen. (c) Frequency and numbers of tetramer binding CD8 (specific to NP396 or GP33 epitopes) and CD4 (specific for GP66 epitope) T cells in the spleen. (d) Splenocytes were stimulated for 5 hrs in vitro with LCMV peptides in the presence of Brefeldin A, and cytokine production was measure by intracellular staining for IFN*γ* in CD8 and CD4 T cells from the spleen. (e) Frequency and numbers of Tregs in the lung of influenza virus-infected mice. (f) Frequency of effector Treg subsets in the lung. (g) Frequency and numbers of influenza virus-specific CD8 (specific to NP66 and PA224 epitopes) and CD4 (specific to NP311 epitope) T cells in the lung. (h) Mononuclear cells from the lung were stimulated for 5 hrs in vitro with PR8 peptides or PMA and ionomycin in the presence of Brefeldin A, and cytokine production was measured by intracellular staining of IFN*γ* in CD8 and CD4 T cells from the lung. This experiment was repeated three times with similar results, 4-5 mice per group. ∗*p* < 0.05.

Viral infections typically trigger Type 1 immune responses and Tregs play a crucial role in restraining the effector phase of T cell responses to mitigate inflammation and the associated tissue damage. Data in Figure 1 showed that conventional CD8 and CD4 T cells from Bach2^loxp/loxp^FoxP3-Cre mice displayed greater activated/effector phenotypic and functional properties, as compared to conventional T cells from WT mice. The elevated levels of IFN*γ* production in CD4 T cells of Bach2^loxp/loxp^FoxP3-Cre mice suggested that deficiency for Bach2 in Tregs might lead to dysregulated Type 1 immunity. Therefore, it was of interest to determine whether (1) dysregulated Treg homeostasis in Bach2^loxp/loxp^FoxP3-Cre mice is altered by viral infections and (2) Bach2 deficiency in Tregs affects the development of T_H_1 and T_C_1 type T cells during an acute viral infection. First, we infected cohorts of WT and Bach2^loxp/loxp^FoxP3-Cre mice with lymphocytic choriomeningitis virus (LCMV) and assessed Tregs in spleen at day 8 after infection. The percentages and total number of Foxp3^+ve^ Tregs in spleens of LCMV-infected Bach2^loxp/loxp^FoxP3-Cre mice were similar to those in WT mice (Figure 4(a)). Next, we evaluated the expression of surface markers associated with an activated/effector Treg phenotype in LCMV-infected WT and Bach2^loxp/loxp^FoxP3-Cre mice. There were no significant changes in the percentages of CD44, CD62L, CXCR3, CD27, CD127, and KLRG-1-expressing Tregs in spleens of Bach2^loxp/loxp^FoxP3-Cre mice. However, greater percentages of Bach2-deficient Tregs expressed CD69 and GITR, as compared to WT Tregs (Figure 4(b)). Thus, overall, as compared to Tregs in uninfected mice (Figure 1), LCMV infection led to activation of WT Tregs, and the levels were similar to those in LCMV-infected Bach2^loxp/loxp^FoxP3-Cre mice.

Next, we assessed the effect of Treg-specific Bach2 deficiency on CD4 and CD8 T cell responses to LCMV infection. The frequency and numbers of LCMV-specific CD8 T cells in Bach2^loxp/loxp^FoxP3-Cre mice were similar to those in WT mice. Likewise, the numbers of CD4 T cells specific to the LCMV GP66 epitope in WT mice were comparable to those in Bach2^loxp/loxp^FoxP3-Cre mice (Figure 4(c)). To investigate if Bach2 deficiency altered cytokine production by LCMV-specific CD4 and CD8 T cells, we measured antigen-induced IFN-*γ* production ex vivo. We did not find differences in the percentages of LCMV-specific IFN-*γ*-producing CD8 and CD4 T cells in spleens between WT and Bach2^loxp/loxp^FoxP3-Cre mice (Figure 4(d)). Taken together, data in Figure 4 suggest that Treg-specific Bach2 deficiency had no detectable effect on the development of CD4 and CD8 T cell responses during an acute LCMV infection.

Next, we assessed the effect of Treg-specific Bach2 deficiency on T_H_1/T_C_1 responses to a mucosal infection with influenza A virus (IAV). First, we infected Bach2^loxp/loxp^FoxP3-Cre and WT mice with PR8 strain of IAV and analyzed Tregs in the lung on day 10 post-infection. We did not find significant differences in the number of Tregs between the Bach2^loxp/loxp^FoxP3-Cre and WT mice in the lung (Figure 4(e)). Likewise, there were no changes in the percentages of CD44, CD62L, CXCR3, CD103, CD27, CD69, CD127, and KLRG-1-expressing Tregs in the lung or BAL of influenza virus-infected Bach2^loxp/loxp^FoxP3-Cre mice (Figure 4(f)).

Next, we investigated whether Bach2-deficiency in regulatory T cells altered the activation and expansion of effector CD8 and CD4 T cells during an influenza infection. No significant differences in the frequencies or total number of influenza-specific CD8 or CD4 T cells were seen in the lung and BAL of Bach2^loxp/loxp^FoxP3-Cre mice (Figure 4(g)). To determine if there were any changes in the production of IFN-*γ* by influenza-specific CD4 and CD8 T cells, we stimulated lymphocytes in the lung with corresponding influenza peptides and measured IFN-*γ* production. Although stimulation with the PA224 peptide elicited greater percentages of IFN-*γ*-producing CD8 T cells in Bach2^loxp/loxp^FoxP3-Cre mice (Figure 4(h)), Bach2-deficiency in Tregs had minimal effects on the activation or cytokine production of CD8 or CD4 T cells, during a mucosal T_H_1/T_C_1 response to influenza virus.

### 3.5. Bach2 Deficiency in Tregs Exacerbates Fungal Protease-Induced T_H_2 Immunity

**Figure 5:**
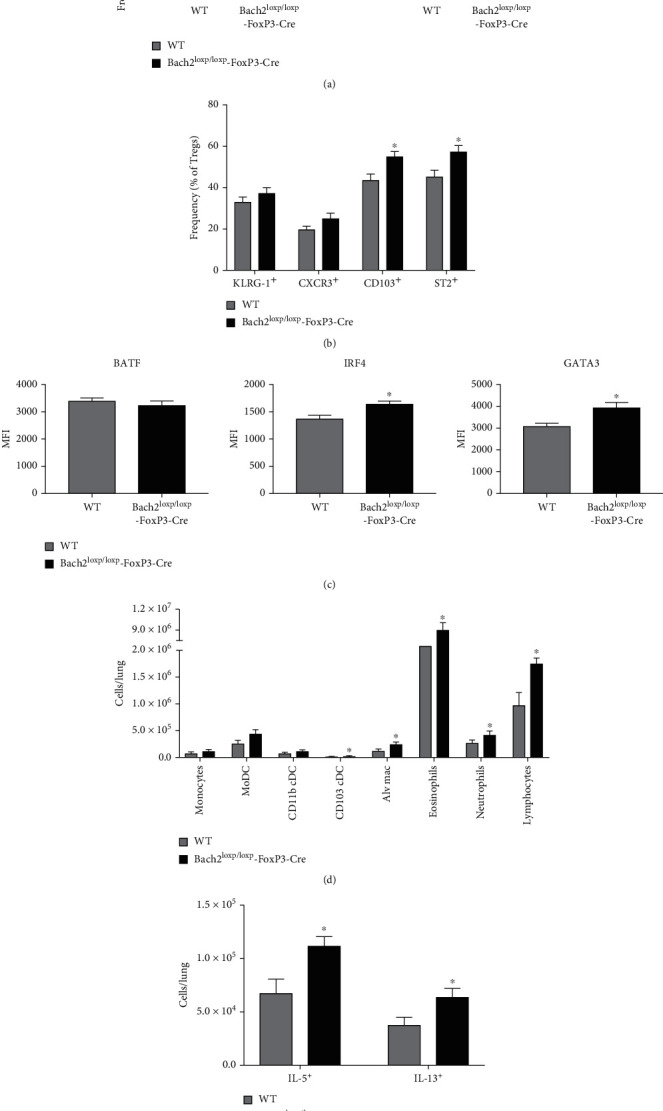
Bach2 is required for Tregs to control T_H_2 immunity in lungs. WT and Bach2^loxp/loxp^FoxP3-Cre mice were administered *Aspergillus melleus* fungal protease intratracheally and lungs were harvested on day 15; mononuclear cells from lungs were analyzed by flow cytometry. (a) Frequency and numbers of Foxp3^+ve^ Tregs in the lungs. (b) Frequency of KLRG-1-, CXCR3-, CD103-, and ST2-positive Tregs in lungs. (c) MFI levels for BATF, IRF4, and GATA3 staining in Foxp3^+ve^ Tregs. (d) Numbers of inflammatory cell subsets in the lungs. (e) Cells from the lung were stimulated for 6 hrs in vitro with heat-inactivated fungal protease in the presence of Brefeldin A during the last 4 hrs, and cytokine production was measure by intracellular staining of IFN*γ*, IL-5, IL-13, and IL17 in CD4 T cells. This experiment was repeated two times with similar results, 4-6 mice per group. ∗*p* < 0.05.

Genome-wide association studies have linked variations in Bach2 gene to asthma in humans, and global Bach2 deficiency in mice leads to unprovoked T_H_2 immunity and fatal eosinophilic crystalline pneumonia [20, 32, 33]. Additionally, Bach2 deficiency in all T cells leads to spontaneous development of T_H_2-driven lung disease [24]. We observed that Treg-specific Bach2 deficiency did not result in overt lung pathology, which suggested that Bach2 expression in conventional T cells is sufficient to protect against spontaneous T_H_2 lung disease. However, it was unknown whether Bach2 deficiency in Tregs affected (1) their responses to allergic inflammation and (2) T_H_2 responses and susceptibility of mice to allergen-provoked inflammation in the lungs. To investigate the effect of Treg-specific Bach2 deficiency on T_H_2 immunity, we utilized a model of allergic inflammation that is induced by administration of alkaline protease 1 of *Aspergillus melleus*; *Aspergillus* protease has been known to elicit a potent T_H_2 response in the lungs [34]. The immunological response of Bach2^loxp/loxp^FoxP3-Cre and WT mice to *Aspergillus* protease administration is shown in Figure 5. As shown in Figure 5(a), protease administration induced substantial increases in the numbers of Tregs in the lungs of WT mice, and protease-induced Treg accumulation was markedly accentuated in lungs of Bach2^loxp/loxp^FoxP3-Cre mice. The increased numbers of Tregs in lungs of protease-treated Bach2^loxp/loxp^FoxP3-Cre mice included CXCR3^+ve^, CD103^+ve^, ST2^+ve^, and KLRG-1^+ve^ Tregs (Figure 5(b)), and this was associated with elevated levels of IRF4 and GATA3 (Figure 5(c)). We also compared the accumulation of inflammatory cells in the lungs of protease-treated WT and Bach2^loxp/loxp^FoxP3-Cre mice (Figure 5(d)). Overall, lungs of protease-treated Bach2^loxp/loxp^FoxP3-Cre mice contained higher numbers of monocytes, monocyte-derived DCs, CD103^+ve^ DCs, inflammatory DCs, alveolar macrophages, neutrophils, and eosinophils, as compared to protease-treated WT mice. Next, we assessed whether Treg-specific Bach2 ablation affected the T_H_1/T_H_2/T_H_17 polarization of protease-reactive CD4 T cells in the lungs. Significantly more IL-5- and IL-13-producing CD4 T cells were detected in the lungs of protease-treated Bach2^loxp/loxp^FoxP3-Cre mice than in WT mice (Figure 5(e)). Taken together, data in Figure 5 strongly suggest that Bach2 plays a critical role in promoting the ability of Tregs to limit T_H_2 immunity and allergic inflammation in the lungs.

## 4. Discussion

It has been established that ST2^+ve^ tissue Tregs are a specialized subset of effector Tregs that reside primarily in non-lymphoid tissues [14, 15], but not in the spleen [15, 16, 35, 36]. In this study, we report a surprising finding that a substantive proportion of Bach2-deficient Tregs in spleen expressed conventional activation markers and elevated levels of tissue Treg markers CXCR3, CD103, and ST2. The increase in the numbers of effector/tissue Tregs in spleen of Treg-specific Bach2-deficient mice cannot be explained by defective homing to the peripheral tissues because lungs and VAT contained normal or greater numbers of Tregs. Apart from IL-2R and TCR signaling, development of eTregs is driven by inflammatory milieu in the peripheral tissues [7–13]. Governed by extracellular cues, transcription factors BATF and IRF4 maintain tissue Tregs, orchestrate the effector Treg transcriptional program, and promote expression of ST2 [15]. Subsequently, IL-33 induces GATA-3 phosphorylation, which binds to the ST2 locus to enhance ST2 gene expression [16]. The expression of ST2 in T_H_2 [37, 38] and tissue Tregs [16, 39] is reliant on a positive feedback loop where IL-33 induces GATA3 recruitment to the ST2 locus, *IL1rl1.* Studies by Vasanthakumar et al. demonstrated that IRF4 and BATF binding to the *IL1rl1* loci is required for maintaining VAT Treg identity, and administration of IL-33 amplifies the number of Tregs in the VAT [15]. Our discovery of ST2^+ve^GATA3^+ve^ Bach2-deficient Tregs in the spleen prompted us to further investigate whether Bach2-deficient splenic Tregs are hypersensitive to IL-33, as compared to their WT counterparts. We find that IL-33 exposure in vitro further increased the expression of ST2 in splenic Bach2-deficient Tregs. This increase is impressive considering that Bach2-deficient Tregs already express elevated basal levels of ST2 compared to WT Tregs. This increase of ST2 expression was associated with increased frequencies of GATA3-, BATF-, and IRF4-expressing cells, which suggest that Bach2 might repress GATA3/BATF/IRF4-driven expression of ST2 and subsequent differentiation of tissue Tregs in the spleen. Furthermore, Bach2 restrains differentiation of eTregs by competing with, or directly repressing BATF, IRF4, and GATA3 expression in response to extracellular cues [21, 24–27], which in turn can limit ST2 expression and eTreg development.

Most notably, Treg phenotypes were largely normal in non-lymphoid tissues such as lungs, but Bach2-deficiency in Tregs resulted in substantive increase in the numbers of CXCR3+ and CD103+ Tregs in the VAT. Although there was not any detectable change of ST2 expression, Bach2-deficient Tregs exhibited higher levels of GATA3 and IRF4, overall suggesting that Bach2 is needed to repress tissue Treg differentiation in the VAT. In the present study, we found that in vivo IL-33 treatment increased the numbers of Tregs in VAT of Bach2^loxp/loxp^FoxP3-Cre and WT mice and that these IL-33-responsive cells were CD103^+ve^, CXCR3^+ve^, and ST2^+ve^ Tregs. Notably, WT Treg levels of GATA3, BATF, and IRF4 were induced to levels that were comparable to those of IL-33-treated Bach2-deficient Tregs. In contrast to our in vitro experiment with splenic Tregs, IL-33 treatment did not induce significant in vivo changes in the splenic Treg population in Bach2-deficient mice. This difference could be attributed to the fact that Bach2-deficient Tregs already demonstrate a higher basal level of expression of tissue Treg phenotype compared to WT and, in vivo, it could take a higher concentration to exacerbate this phenotype than the regimen that we used. Alternatively, IL-33-induced Tregs in spleen might have already re-localized to VAT.

Despite displaying a robust effector phenotype, Bach2-deficient Tregs seemed to be incapable of restraining the activation of conventional T cells. Higher numbers of CD8 and CD4 T cells in Treg-specific Bach2-deficient mice exhibited an activated/effector phenotype as determined by CD44 and CD62L expression. Further, these CD8 and CD4 T cells secreted substantial amounts of pro-inflammatory cytokines. Specifically, higher percentages of CD4 T cells from Treg-specific Bach2-deficient mice secrete IFN*γ*, IL-13, and IL-17A, suggesting that Tregs require Bach2 expression to repress the activation and differentiation of T_H_1, T_H_2, and T_H_17 cells, respectively. These findings led us to explore whether Bach2-deficient Tregs are unable to control Type 1 and Type 2 responses in vivo. Acute viral infections typically induce Type 1 responses, and it was of interest to assess whether Bach2 deficiency altered the effect of LCMV infection on Tregs and whether Treg-specific Bach2 deficiency led to enhanced T cell responses to an LCMV infection. Surprisingly, Treg-specific Bach2 deficiency has no detectable effect on the numbers of Tregs or the magnitude of virus-specific CD8/CD4 T cell response or the cytokine-producing ability of virus-specific CD8/CD4 T cells. Unlike in an LCMV infection, Treg-specific Bach2 deficiency limits the expansion of Tregs in mice infected with *Plasmodium chabaudi* [26]. Thus, Bach2 regulation of Tregs depends on the infection type. We also found that Treg-specific Bach2 deficiency had minimal effects on mucosal T cell responses in the lungs during an acute influenza virus infection. Notably, in a model of chemically-induced colitis, loss of Bach2 in Tregs protected against colitis [26]. An emerging theme from our studies is that loss of Bach2 expression in Tregs does not affect T_H_1 responses to systemic or mucosal viral infections.

We have previously reported that global knockout of Bach2 leads to the development of Type 2 cytokine-driven fatal eosinophilic crystalline pneumonia [20]. Further, loss of Bach2 in all T cells also leads to aberrant T_H_2 immunity and lung disease [24]. Treg-specific Bach2 deficiency did not lead to the unprovoked development of eosinophilic crystalline pneumonia or T_H_2-driven lung disease for at least until 8-9 months of age. Thus, Bach2 deficiency in Tregs is not sufficient to cause unprovoked T_H_2-driven lung inflammation. Since variations in the Bach2 gene have been linked to asthma in humans and Bach2 deficiency led to eosinophilic crystalline pneumonia in mice [20, 32, 33], we assessed whether Treg-specific Bach2 deficiency affected the development of fungal protease-induced allergic inflammation in the lungs. Recent work has shown that some allergen proteases possess the ability to cleave and potentiate IL-33 [40, 41]. IL-33 is then capable of driving an ST2-dependent type 2 inflammatory response. Remarkably, despite expressing high basal levels of GATA3 and ST2, Bach2-deficient Tregs appeared to be unable to suppress *Aspergillus* protease-induced allergic inflammation. Bach2-deficient Tregs expressed a more activated phenotype with protease treatment, and these increases were paralleled by increases of effector phenotypes in WT counterparts as well. Overall, these data suggest that Bach2 is required for Tregs to restrain T_H_2 inflammation. Mechanistically, the absence of Bach2 leads to increased levels of GATA3, BATF, and IRF4, and subsequently more ST2 expression. The inherently higher basal level of an activated/effector tissue Treg phenotype in Bach2-deficient Tregs makes it difficult to detect further activation or display of effector characteristics; however, it should be noted that WT Tregs need to be stimulated in order to reach the basal level of magnitude that Bach2-deficient Tregs express.

## 5. Conclusions

Maintenance of Treg homeostasis and effector function in lymphoid and nonlymphoid tissues is critical for mitigating autoimmunity and inflammatory diseases. In this manuscript, we ascribe vital roles for Bach2 in regulating the numbers and functions of effector Tregs in lymphoid and non-lymphoid tissues. First, we confirm previous findings [25–27] that Treg-specific Bach2 deficiency leads to unprovoked precocious differentiation of effector Tregs in lymphoid tissues. Second, we document that Bach2 restrains the development of CXCR3 + CD103 + ST2+ tissue Tregs in secondary lymphoid tissues. Third, we show that Bach2 in Tregs limits the number of CXCR3 + CD103+ Tregs in VAT. Fourth, loss of Bach2 in Tregs does not affect Type 1 immunity to systemic and mucosal viral infections. Fifth, we show that Treg-specific Bach2 deficiency does not result in unprovoked T_H_2-driven inflammation, but restrains aggressive fungus protease-induced Type 2 inflammation in lungs by functioning as a transcriptional checkpoint in both Tregs and conventional effector cells. In summary, findings presented in this manuscript provide mechanistic insights into the role of Bach2 in regulating Treg homeostasis and protecting humans against Type 2 immunity such as asthma and other allergic disorders.

## Data Availability

All data presented in this manuscript is archived in secure computers at the University of Wisconsin-Madison.
